# Native cell-death genes as candidates for developing wilt resistance in transgenic banana plants

**DOI:** 10.1093/aobpla/plu037

**Published:** 2014-07-04

**Authors:** Siddhesh B. Ghag, Upendra K. Singh Shekhawat, Thumballi R. Ganapathi

**Affiliations:** Plant Cell Culture Technology Section, Nuclear Agriculture and Biotechnology Division, Bhabha Atomic Research Centre, Trombay, Mumbai 400 085, India

**Keywords:** Banana, disease resistance, Fusarium wilt, PCD, transgenic, UPR.

## Abstract

In order to feed an ever-increasing world population, there is an urgent need to improve the production of staple food and fruit crops. The productivity of important food and fruit crops is constrained by numerous biotic and abiotic factors. The cultivation of banana, which is an important fruit crop, is severely threatened by Fusarium wilt disease caused by infestation by an ascomycetes fungus *Fusarium oxysporum* f. sp. *cubense* (Foc). Since there are no established edible cultivars of banana resistant to all the pathogenic races of Foc, genetic engineering is the only option for the generation of resistant cultivars. Since Foc is a hemibiotrophic fungus, investigations into the roles played by different cell-death-related genes in the progression of Foc infection on host banana plants are important. Towards this goal, three such genes namely *MusaDAD1*, *MusaBAG1* and *MusaBI1* were identified in banana. The study of their expression pattern in banana cells in response to Foc inoculation (using Foc cultures or fungal toxins like fusaric acid and beauvericin) indicated that they were indeed differentially regulated by fungal inoculation. Among the three genes studied, *MusaBAG1* showed the highest up-regulation upon Foc inoculation. Further, in order to characterize these genes in the context of Foc infection in banana, we generated transgenic banana plants constitutively overexpressing the three genes that were later subjected to Foc bioassays in a contained greenhouse. Among the three groups of transgenics tested, transformed banana plants overexpressing *MusaBAG1* demonstrated the best resistance towards Foc infection. Further, these plants also showed the highest relative overexpression of the transgene (*MusaBAG1*) among the three groups of transformed plants generated. Our study showed for the first time that native genes like *MusaBAG1* can be used to develop transgenic banana plants with efficient resistance towards pathogens like Foc.

## Abstract (precis)

Banana is the fourth most important food commodity of the world and forms the staple food of majority of people in the tropical and subtropical regions. Banana production is severely constrained by Fusarium wilt disease that causes enormous loss. The present study developed transgenic banana plants overexpressing native cell death genes to impart Fusarium wilt resistance. Since banana is predominantly a vegetatively propagated crop, genetic engineering is the most viable option for development of resistance against important diseases of this crop.

## Introduction

By the year 2050, the world's population is expected to be double the present number, making food security for all the most important social issue over the next 35 years. To feed this surge in the population, the annual food output will have to be at least doubled in future. To address this enormous task, agricultural productivity should be increased in a sustainable way. The most important constraints in achieving this target are the numerous biotic stress factors that cumulatively contribute to a significant lowering of the productivity of important food and fruit crops to below what their actual genetic yield potential can confer. Banana is counted among the most important food security crops in the world after rice, wheat and maize. Although the potential of banana as a cash and export commodity is generally considered to be huge, diseases and pests have hindered efforts towards increasing its production in the last few decades (Jones 2009). *Fusarium oxysporum* f. sp. *cubense* (Foc), the causative agent of Fusarium wilt disease of banana (Ploetz 2006), is a hemibiotrophic fungus that infects the root system of the banana plant. Historically, Fusarium wilt has been the major biotic constraint in all banana-growing regions of the world. Once the fungal mycelia enters the roots through natural injuries, the rapidly multiplying fungal mass occludes the xylem vessels leading to insufficient supply of nutrients and water to the plant and causing yellowing of the leaves and eventually wilting of the whole plant (Li *et al.* 2011). Among the known races of Foc, Race 1 and Race 4 are of major concern internationally to the banana industry. Spread of Foc in the 19th century resulted in shifting of mass cultivation to Foc Race 1 resistant Cavendish varieties throughout the world. To date, the Cavendish varieties have held an eminent position in the global market. But, owing to the popularity of a select few Cavendish cultivars in major banana-producing areas of the world, the threat of a debilitating disease epidemic is very real in banana. Spread of highly virulent Cavendish infecting Foc Race 4 (Buddenhagen 2009) beyond South-East Asia and Australia (Butler 2013) is all the more worrisome as there are no established Foc Race 4 resistant cultivars [although several research groups have claimed recovery of somaclonal variants showing promising resistance towards Foc Race 4 (Hwang and Ko 2004)]. Owing to their complex ploidy and parthenocarpic fruit development, conventional breeding is laborious and time consuming in edible cultivars of banana and hence introduction of resistance traits into elite Cavendish varieties through breeding seems to be a distant, if not impossible, goal (Tang 2005; Heslop-Harrison and Schwarzacher 2007). Genetic modification of banana through appropriate strategies is potentially the best approach for developing elite edible banana plants resistant to all races of Foc. Among the non-Cavendish varieties that are susceptible to Foc Race 1 as well as Foc Race 4, cv. *Rasthali* (AAB ‘Silk’ group) is a highly prized banana cultivar preferred for its unique fruity aroma and golden yellow colour. Introduction of Fusarium wilt resistance in cv. *Rasthali* therefore remains an important objective.

Diverse pathogenic fungi belonging to *F. oxysporum* have a short biotrophic phase inside the host plant which is followed by complete necrotrophy of the infected plant (Thaler *et al.* 2004). Induction of necrotrophy in the infected plant probably involves activation of the plant cell death (PCD) process (Lam *et al.* 2001; Dickman and de Figueiredo 2013). Therefore, for infections involving necrotrophic or hemibiotrophic fungi, modulating the level of genes which negatively regulate the PCD pathway of the host plant may provide insight into their role in disease progression. Also, overexpression of these genes in the transgenic plants would probably lead to prevention of the cell death occurring due to this kind of pathogen attack. In fact, transgenic tobacco plants overproducing chicken Bcl-X_L_ proteins, nematode CED-9 and baculovirus Op-IAP have been shown to resist a multitude of pathogens by suppressing cell death (Mitsuhara *et al.* 1999; Dickman *et al.* 2001).

Perception of a fungal pathogen attack in plants causes heightened nucleocytoplasmic activity leading to expression of mainly secretory proteins targeted against the invading pathogen. During this response the number of proteins that accumulate in the endoplasmic reticulum (ER) goes beyond its capacity, hence several of them are left misfolded or unfolded, causing an unfolded protein response (UPR) ultimately leading to PCD (Liua and Howell 2010). Among the well-studied PCD-associated proteins in plants, Defender Against Death domain (DAD) protein is known to undertake a dedicated step in glycosylation of proteins (vital for their correct folding and activity) as it codes for a subunit of oligosaccharyltransferase that transfers a high mannose oligosaccharide to the asparagine residue of the polypeptides in the rough ER. Sequences coding for DAD have been widely reported to be involved in cell death and senescence in plants such as *Arabidopsis*, rice and pea (Dong *et al.* 1998). Other such proteins are the Bcl-2-associated athanogenes (BAG) which function as co-chaperones in diverse physiological roles in cell survival, cell differentiation and stress responses (Doukhanina *et al.* 2006). *Arabidopsis* BAG7 protein (AtBAG7) is an ER resident protein which takes part in the UPR response, thereby assisting in restoration of cellular homoeostasis by facilitating efficient protein folding (Williams *et al.* 2010). In contrast, Bcl-2-associated X protein (BAX) is a pro-apoptotic protein that forms channels in the mitochondrial outer membrane thereby releasing cytochrome *c* which activates the caspase-mediated cell death process in animal cells. BAX inhibitor (BI) is a membrane-bound protein that blocks the activity of BAX protein by an unknown mechanism leading to the suppression of cell death processes induced during fungal infections, by fungal toxins, salicylic acid, reactive oxygen species and during ER stress (Hückelhoven *et al.* 2003; Watanabe and Lam 2008). *BI-1* (*OsBI-1*) gene was shown to be down-regulated in rice suspension cells upon treatment with the cell wall extract of rice blast fungus *Magnaporthe grisea* (Matsumura *et al.* 2003). Further, the transgenic rice cells overexpressing *Arabidopsis BI-1* (*AtBI-1*) gene showed resistance to cell death caused by the above-mentioned elicitor treatment. BAX inhibitor-1 (TaBI-1) in wheat was identified as a negative cell death regulator and conferred resistance against stripe rust (Wang *et al.* 2012). On infecting the host plant necrotophic fungi create a chronic stress condition leading to induction of the UPR response that finally results in cell death (Williams *et al.* 2010).

It still remains unclear whether Foc causes cell death in banana tissues by deposition of potent fungal toxins like fusaric acid (FA) or beauvericin (BEA) or simply by inducing host cell death initiating proteins thereby leading to PCD. A recent report illustrated that overexpression of *Bcl-xL*, *Bcl-2* 3′ UTR and *Ced-9* transgenes in transgenic banana plants conferred resistance against Foc Race 1 (Paul *et al.* 2011). Consequent upon the results of the above-mentioned study, we postulated that constitutive overexpression of the native genes coding for similar cell-death-related proteins in transgenic banana plants can impart resistance to pathogenic fungi like Foc. In the present study, we identified genes coding for three such proteins from banana EST databases and subsequently overexpressed them in transgenic banana plants to analyse whether their overexpression could provide efficient resistance against Foc in these transgenic banana plants.

## Methods

### Primers

Primers used in the present study are listed in **Supporting Information**.

### PCR amplification and sequence analysis

Total RNA was extracted from young banana (cv. *Rasthali*) leaves using Concert Plant RNA Reagent and RNeasy Plant Mini Kit as described above. Accuscript Reverse Transcriptase was used to make cDNA from this total RNA. Full length coding sequences of *MusaDAD1*, *MusaBAG1* and *MusaBI1* were amplified from the cDNA prepared as above using Pfu Ultra AD DNA Polymerase (Agilent Technologies, USA) and subsequently sequenced. Predicted protein sequences of three genes cloned were aligned with their closest homologues using ClustalW2 program (http://www.ebi.ac.uk/Tools/clustalw2/index.html). Evolutionary relationships of the three genes were studied using a MEGA 5 software tool.

### Expression profiling of major cell-death-related genes in banana suspension culture cells in response to Foc Race 1 inoculation

In order to ascertain the expression pattern of the three identified cell-death-related genes in response to Foc inoculation in banana, suspension culture cells of banana (cv. *Rasthali*) maintained in M2 medium (Ganapathi *et al.* 2001) were treated with actively growing Foc Race 1 culture (aFoc) grown for 4 days in potato dextrose broth (PDB) and used at a final concentration of 0.5 % v/v and double autoclaved filtrate of the same culture (fFoc) at a final concentration of 0.5 % v/v (Yoshikawa *et al.* 1981). In addition, the banana cells were also treated with two known *Fusarium* toxins namely FA (at a final concentration of 50 μM) and BEA (at a final concentration of 5 μM). Total RNA was extracted from the treated as well as control untreated cells using Concert Plant RNA Reagent (Invitrogen, USA). The extract obtained after chloroform extraction was cleaned and treated with DNase using the RNeasy Plant Mini Kit (Qiagen, Germany). This RNA (∼5 μg) was then used to make first-strand cDNA using Oligo (dT)_12–18_ primer (Invitrogen) and AccuScript Reverse Transcriptase (Stratagene, USA) following the manufacturer's instructions. Three independently treated cell aliquots for each treatment were pooled prior to RNA isolation to ensure reproducibility of results. cDNAs derived from the treated and untreated controls were then diluted appropriately and used for quantitative real-time RT–PCR using SYBR Green Extract-N-Amp PCR ReadyMix (Sigma, USA) and Rotor-Gene Q platform (Qiagen). Primers specific for the three identified genes (*MusaDAD1*, *MusaBAG1* and *MusaBI1*) were used in these reactions alongside primers specific for *Musa acuminata elongation factor 1α (EF1α)* coding sequence so as to allow gene expression normalization and subsequent quantification. *C*_t_ values obtained using the Rotor-Gene software were analysed using REST-MCS utility (Pfaffl *et al.* 2002) to analyse the expression patterns of the three genes named above in response to Foc inoculation.

### Construction of plant expression vectors

Sequenced coding sequences of the three genes were cloned into the multiple cloning site of the pCAMBIA-1301 plant expression vector together with *Zea mays* polyubiquitin promoter and nopaline synthase terminator in a three-way ligation reaction as described previously (Shekhawat *et al.* 2011; Ghag *et al.* 2012; Sreedharan *et al.* 2013). Newly constructed binary vectors named p1301-*MusaDAD1*, p1301-*MusaBAG1* and p1301-*MusaBI1* were subsequently mobilized into *Agrobacterium tumefaciens* strain EHA 105 by electroporation for use in genetic transformation.

### Generation of transgenic banana lines and genomic DNA PCR analysis

Embryogenic cell suspension cultures of banana cv. *Rasthali* were utilized for *Agrobacterium*-mediated genetic transformation using the three newly constructed binary vectors as essentially described previously (Ganapathi *et al.* 2001). Putatively transgenic hardened banana plants were examined for their transgenic character by genomic DNA PCR. Genomic DNA was isolated from young leaves of transformed and untransformed banana plants using the GenElute Plant Genomic DNA Miniprep Kit (Sigma). These were then used in PCR reactions along with primers specific to *hygromycin phosphotransferase* coding sequence to ascertain the presence of T-DNA in the putatively transgenic banana plants. Selected plants were also analysed by PCR using primers specific for the T-DNA region and designed to amplify the respective coding sequence and the nos 3′ UTR region.

### Screening of transformants for resistance to Foc Race 1 infection

Seven-day-old culture of Foc Race 1 grown on potato dextrose agar (PDA) was inoculated in PDB medium and allowed to grow at 30 ± 1 °C for 5 days. The fungal spores were separated from the mycelium by sieving the culture through four layers of cheese cloth. The spores were washed with sterile distilled water and adjusted to a spore density of 8 × 10^5^ spores mL^−1^. Further, the fungal mass culture was prepared by adding the spore suspension to the autoclaved mixture of sand and maize bran (19 : 1) and incubating it at room temperature for 4 weeks. Two-month-old greenhouse acclimatized transgenic banana plants derived from independent transformation events were replanted in the mixture of soil and fungal mass culture in a 1 : 1 ratio and kept under greenhouse conditions (Ghag *et al.* 2012). Foc Race 1 infection, symptoms were observed 6 weeks post-infection. For untransformed control plants and each of the transgenic lines derived from the three constructs, a minimum of four replicates were screened for Foc Race 1 infection and the assays were repeated four times. The plants assayed were scored on a disease severity scale of 1 to 6 (where 1 = healthy, no symptoms; 2 = marginal yellowing and wilting of the lower leaves; 3 ≤ 50 % of leaves yellowing and wilting, <50 % pseudostem cracking; 4 ≥ 50 % of leaves yellowing and wilting, >50 % pseudostem cracking and discolouration, partial discolouration of corm; 5 = extensive yellowing, cracking and discolouration of corm; 6 = complete wilting of the plant). All the plants assayed in the four sequential bioassays were given scores based on this scale according to their symptoms. Subsequently, the mean score of plants derived from a particular line in a bioassay was estimated followed by the calculation of the group mean (denoting the overall performance of a particular transgenic line in the four bioassays conducted). Standard deviation of this group mean was calculated and finally all the group means along with their SD values were plotted to show the results of all the bioassays in totality (Ghag *et al.* 2014). For each of the three constructs, five best performing transgenic banana lines were chosen for further analysis and were photographed along with untransformed controls.

### Southern blot analysis

Genomic DNA was isolated from young leaves of the selected transgenic lines as well as untransformed controls as described previously. Genomic DNAs (∼20 µg) were digested overnight with HindIII restriction enzyme at 37 °C. Digested DNAs were separated overnight at a field strength of 1.25 V cm^−1^ in a 0.9 % (w/v) agarose TAE gel. DNA was then transferred on to a positively charged nylon membrane by a capillary method using 20× SSC buffer. Transferred DNA was UV crosslinked and subsequently immobilized by baking at 120 °C for 30 min. Restricted and immobilized genomic DNA was then allowed to hybridize with DIG-labelled probes targeted against a *hygromycin phosphotransferase* coding sequence present towards the left border in T-DNA of the three binary vectors used to transform banana cells. Chemiluminescent detection of hybridization signals was subsequently carried out in a chemiluminescence detector enabled gel documentation system using DIG High Prime DNA Labeling and Detection Starter Kit II (Roche Applied Science, Germany) according to the manufacturer's instructions.

### Real-time quantitative RT–PCR

In order to estimate the exact quantum of overexpression of the three cell-death-related genes in the respective transgenic banana lines, quantitative RT–PCRs were performed. Total RNA isolation from the transformed and untransformed control leaves and subsequent first-strand cDNA synthesis were performed as described previously. Leaf samples derived from three independent plants of each line were used to isolate the total RNA. cDNAs derived from the three groups of transgenic plants together with untransformed controls were diluted appropriately and then used for quantitative real-time RT–PCR using SYBR Green Extract-N-Amp PCR ReadyMix (Sigma) as described above. *Musa acuminata elongation factor 1α (EF1α)* gene was also amplified along with the three overexpressed genes to allow gene expression normalization and subsequent quantification. *C*_t_ values obtained using the Rotor-Gene software were analysed using REST-MCS utility (Pfaffl *et al.* 2002) to estimate the fold-value expression change of the three overexpressed genes in the respective transgenic plants.

## Results

### Sequence analysis of major cell-death-related genes from banana

Based on the nomenclature of their closest homologues the three genes identified as part of this study were named *MusaDAD1*, *MusaBAG1* and *MusaBI1*. Their coding sequences were amplified using leaf derived cDNA of banana cv. *Rasthali* and they were subsequently sequenced to confirm the presence of the complete ORF. The 348 bp CDS of *MusaDAD1* codes for a 115 amino acid long protein have three putative transmembrane domains similar to its close homologues **[see Supporting Information]**. Alignment with these homologues shows that the domain region is highly conserved among varied plants, implying functional conservation also. The 459 bp amplified CDS of *MusaBAG1* codes for a 152 amino acid long protein have a highly conserved 71 amino acid long ubiquitin-like domain **[see Supporting Information]**. Apart from this domain the close homologues of MusaBAG1 showed low homology, especially in the N-terminal region. The 750 bp coding region of *MusaBI1* gene encodes a protein of 249 amino acids having a 212 amino acid long Bax inhibitor domain **[see Supporting Information]**. Like MusaDAD1, MusaBI1 also had high levels of homology with its close relatives, except at the N-terminal.

### Expression profiling of major cell-death-related genes in banana suspension culture cells

**Figure 1.**
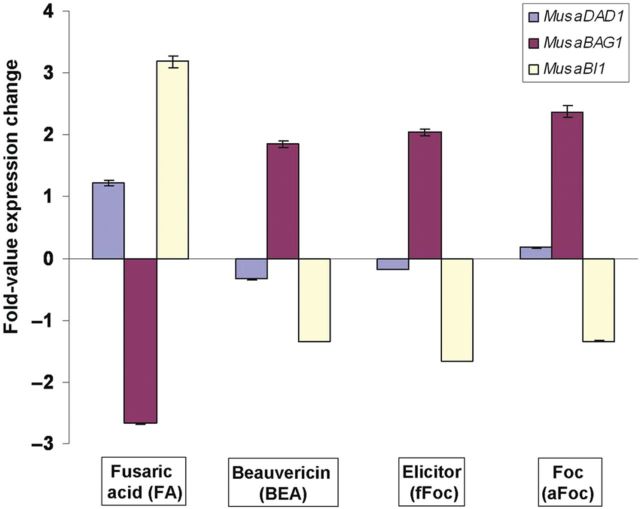
Expression pattern of *MusaDAD1*, *MusaBAG1* and *MusaBI1* in banana (cv. *Rasthali*) embryogenic cell suspension cultures in response to fungal toxins (FA and BEA), active Foc (aFoc) and autoclaved filtrate of Foc (fFoc) 6 h post-treatment determined by quantitative real-time RT–PCR. The *x*-axis represents the expression level of native *MusaDAD1*, *MusaBAG1* and *MusaBI1* in cell suspension cultures. Values are the mean ± SE.

Given that *F. oxysporum* has been accepted to have a hemibiotrophic mode of nutrition during its presence on the plant host, the roles of different host PCD related genes during the infection and colonization process assume importance. Thus, we studied the differential regulation of the three identified PCD related genes (*MusaDAD1*, *MusaBAG1* and *MusaBI1*) in banana in response to Foc infection using two *Fusarium* toxins namely FA and BEA or by utilizing the active Foc culture (aFoc) or an autoclaved filtrate of Foc culture (fFoc). For the three genes studied in this way, striking similarities were noticed in the expression pattern obtained using either BEA, aFoc or fFoc (Fig. 1). In comparison, the expression pattern obtained using FA was completely inverse of what was obtained using the other three treatments. Obviously, this reflected the fact that FA works in a manner which is very different from the other three treatments. Further, *MusaBAG1* showed the highest differential regulation (up-regulation with BEA, aFoc or the fFoc) in our studies, possibly indicating a vital role for this gene in the response of banana to Foc invasion.

### Construction of plant expression vector and generation of transgenic banana plants

**Figure 2.**
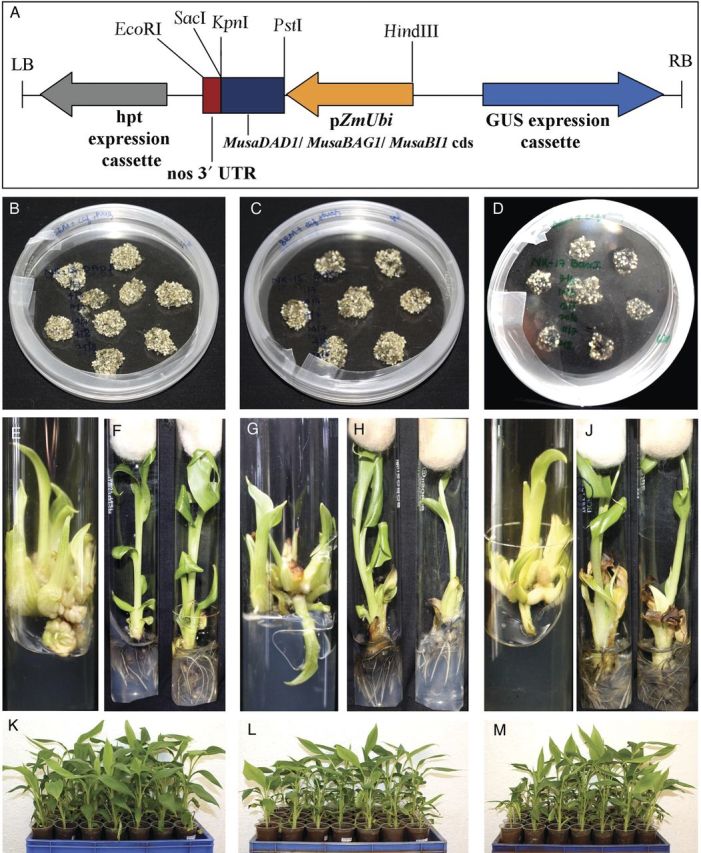
Generation of transgenic banana (cv. *Rasthali*) plants overexpressing *MusaDAD1*, *MusaBAG1* and *MusaBI1*. (A) T-DNA region of the binary vectors p1301-*MusaDAD1*, p1301-*MusaBAG1* and p1301-*MusaBI1* designed to constitutively overexpress the three genes (*MusaDAD1*, *MusaBAG1* and *MusaBI1*) in transgenic banana plants. Putatively transformed embryos derived from p1301-*MusaDAD1* (B), p1301-*MusaBAG1* (C) and p1301-*MusaBI1* (D) selected on hygromycin-based embryo induction medium. Multiple shoots derived from each of the constructs namely p1301-*MusaDAD1* (E), p1301-*MusaBAG1* (G) and p1301-*MusaBI1* (I) on multiple shoot induction medium followed by rooting of the transgenic shoots each of p1301-*MusaDAD1* (F), p1301-*MusaBAG1* (H) and p1301-*MusaBI1* (J) on rooting medium. Rooted plantlets of p1301-*MusaDAD1* (K), p1301-*MusaBAG1* (L) and p1301-*MusaBI1* (M) hardened in a greenhouse (2 months old).

Coding sequence of the three genes were cloned into the MCS region of pCAMBIA-1301 plant expression vector between the *Zea mays* polyubiquitin promoter and nos 3′ UTR to obtain p1301-*MusaDAD1*, p1301-*MusaBAG1* and p1301-*MusaBI1* binary vectors, respectively (Fig. 2A). Embryogenic cell suspension cultures of the banana cv. *Rasthali* were independently transformed with these vectors using the *A. tumefaciens* strain EHA105. Putatively transformed embryos (Fig. 2B–D) were grown in medium supplemented with hygromycin and then germinated under the same selection pressure. The germinated embryos were transferred to a banana multiplication medium for generating multiple shoots of each transformation event (Fig. 2E, G and I). The putatively transformed shoots were rooted (Fig. 2F, H and J) and acclimatized in a greenhouse for 2 months (Fig. 2K–M).

In total, 38, 36 and 33 independently transformed lines were regenerated each from p1301-*MusaDAD1*, p1301-*MusaBAG1* and p1301-*MusaBI1* derived transgenic tissues. The transgenic lines derived from the three vectors did not show any phenotypic abnormalities with respect to the untransformed control banana plants. All the plants so obtained were checked for their transgenic character by carrying out genomic DNA PCR using primers specific for *hygromycin phosphotransferase* coding sequence. A 788 bp band was amplified from all the transgenic lines whereas no amplification was observed in genomic DNA derived from untransformed control plants.

### Foc Race 1 resistant bioassay

**Figure 3.**
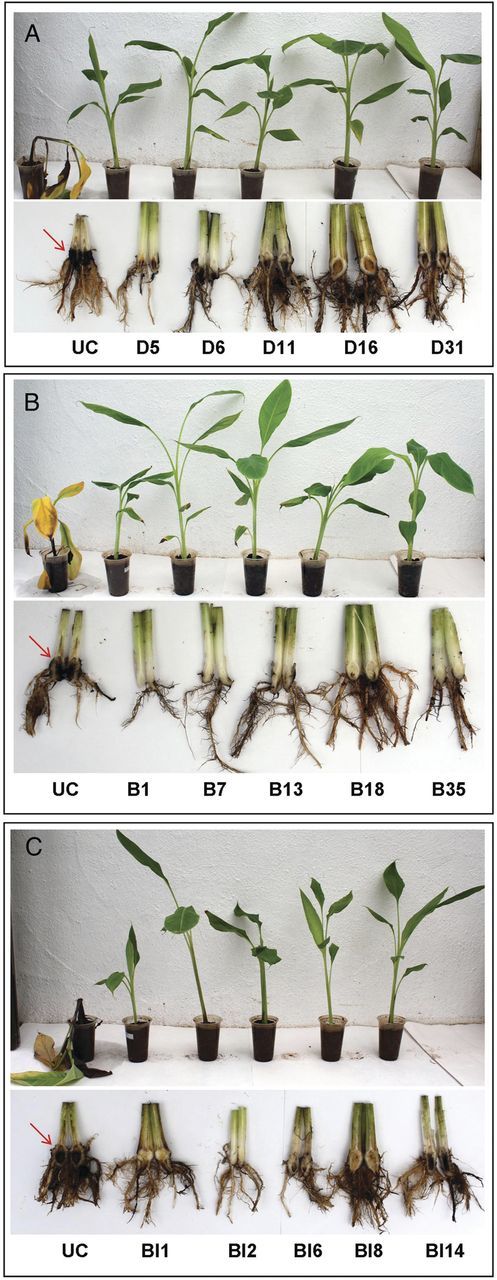
Foc-resistant bioassay of the transgenic banana plants. Two-month-old greenhouse hardened plants were inoculated with the Foc mass culture. Six weeks post-inoculation plants were observed for wilt symptom development. Untransformed control (UC) plants collapsed after 6 weeks due to wilt symptoms whereas transgenic banana plants derived from p1301-*MusaDAD1* (A), p1301-*MusaBAG1* (B) and p1301-*MusaBI1* (C) showed enhanced resistance to Foc. All the plants were cut longitudinally and observed for infestation of Foc. Untransformed control plants showed intense discolouration of the corm due to Foc growth (pointer arrows) as a result of which the plants wilted.

**Figure 4.**
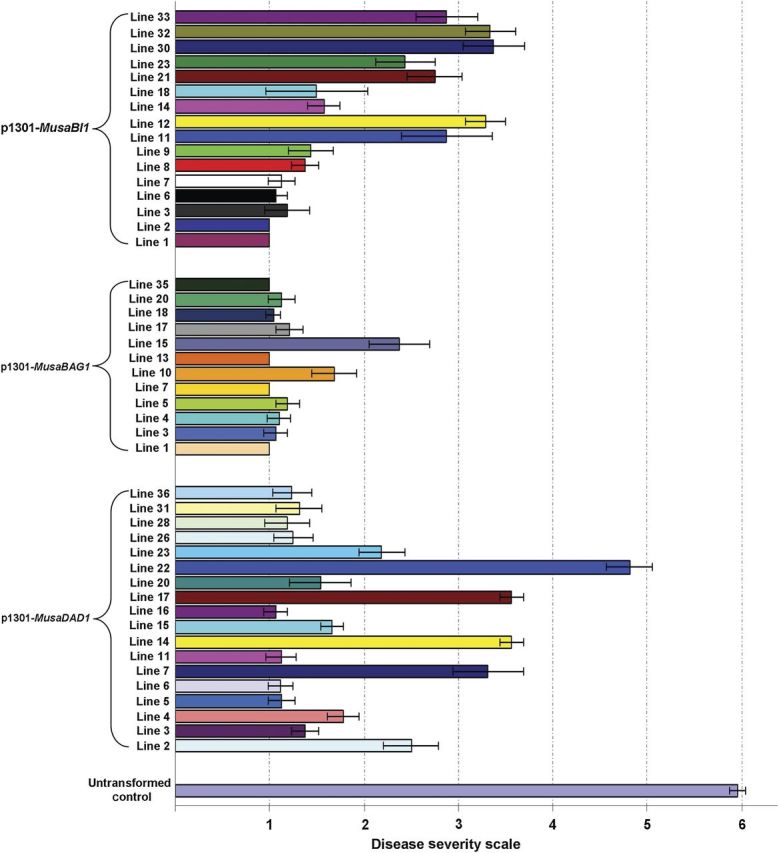
Quantitative disease severity assay for 18 lines derived from p1301-*MusaDAD1* construct, 12 lines derived from p1301-*MusaBAG1* construct and 16 lines derived from p1301-*MusaBI1* construct together with untransformed controls. Five lines derived each from p1301-*MusaDAD1* construct (D5, D6, D11, D16, D31), p1301-*MusaBAG1* construct (B1, B7, B13, B18, B35) and p1301-*MusaBI1* construct (BI1, BI2, BI6, BI8, BI14) were selected for detailed molecular analysis by Southern blotting and real-time RT–PCR.

When 2-month-old hardened transgenic banana plants derived from the three constructs were subjected to Fusarium wilt bioassay using Foc Race 1 cultures, marked Fusarium wilt symptoms were seen in untransformed banana plants at 4 weeks from inoculation. The lower older leaves showed marked yellowing along with complete pseudostem splitting at 6 weeks from infection. Further, there was complete discolouration of the corm from inside (Fig. 3). Among the three groups of transgenic plants derived from the three constructs, plants derived from p1301-*MusaBAG1* were generally healthy with no clear symptoms of Fusarium wilt barring a few lines where we could see mild to moderate symptoms (Fig. 4). In our view, this differential resistance was probably due to different levels of overexpression of the *MusaBAG1* gene in the different independently transformed transgenic lines. Among the other two transgenic groups, some of the lines performed quite clearly better in the bioassays as compared with others.

### Molecular analysis: Southern blotting and quantitative real-time RT–PCR

**Figure 5.**
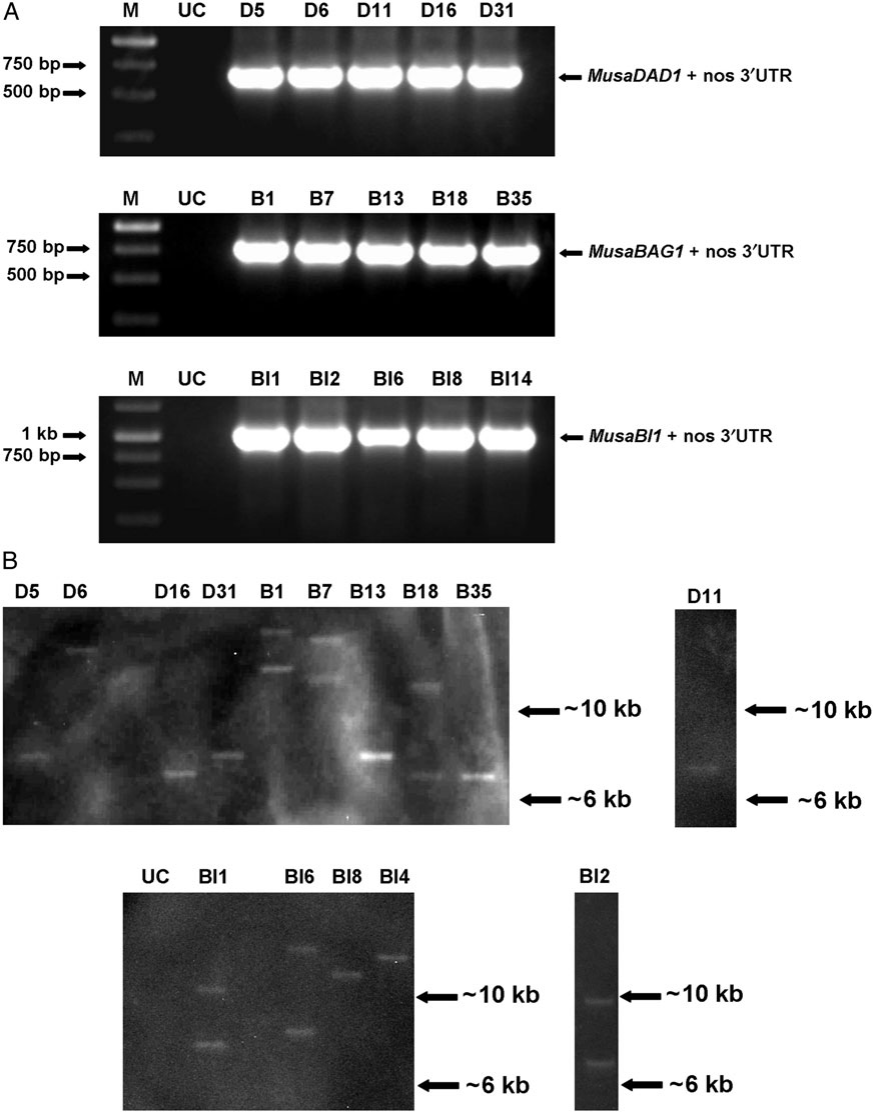
Polymerase chain reaction and Southern blot analysis of transgenic banana plants. (A) Genomic DNA isolated from the five selected transgenic lines derived from each construct was used in PCR reactions using primers specific for T-DNA region of the respective binary vectors. A single amplified product corresponding to the respective gene along with the nos 3′ UTR region was obtained in genomic DNAs derived from transformed banana plants whereas the same were absent in untransformed control (UC) plants. (B) Genomic DNA isolated from young leaves of transformed plants and untransformed control plants was restricted with HindIII and allowed to filter hybridize with DIG-labelled probes against *hygromycin phosphotransferase* coding sequence. The hybridization signal was detected using a chemiluminescence enabled gel documentation system. One to two bands were seen in p1301-*MusaDAD1* (D5, D6, D11, D16, D31), p1301-*MusaBAG1* (B1, B7, B13, B18, B35) and p1301-*MusaBI1* (BI1, BI2, BI6, BI8, BI14) derived transgenic banana plants whereas no hybridization signal was detected in UC.

To further confirm the transgenic nature of the transformed banana plants and to determine the T-DNA copy numbers in the transgenic banana plants, a few plants derived from each of the constructs, which performed best in the bioassays described above, were analysed by genomic DNA PCR using primers specific for the T-DNA region and Southern blotting using probes directed against *hygromycin phosphotransferase* gene carried on the T-DNA. A single amplified product corresponding to the respective gene along with the nos 3′ UTR region was obtained in genomic DNAs derived from transformed banana plants whereas the same were absent in untransformed control plants (Fig. 5A). All the transgenic lines analysed by Southern blotting showed the presence of T-DNA inserted into the banana genome (Fig. 5B). The bands observed could be directly correlated to the number of T-DNA copies present in each of the transgenic lines as the enzyme used to restrict the genomic DNAs was expected to cut the T-DNA only once. The presence of chemiluminescent bands at different positions in different lanes confirmed that all the transgenic plants were derived from independent transformation events.

**Figure 6.**
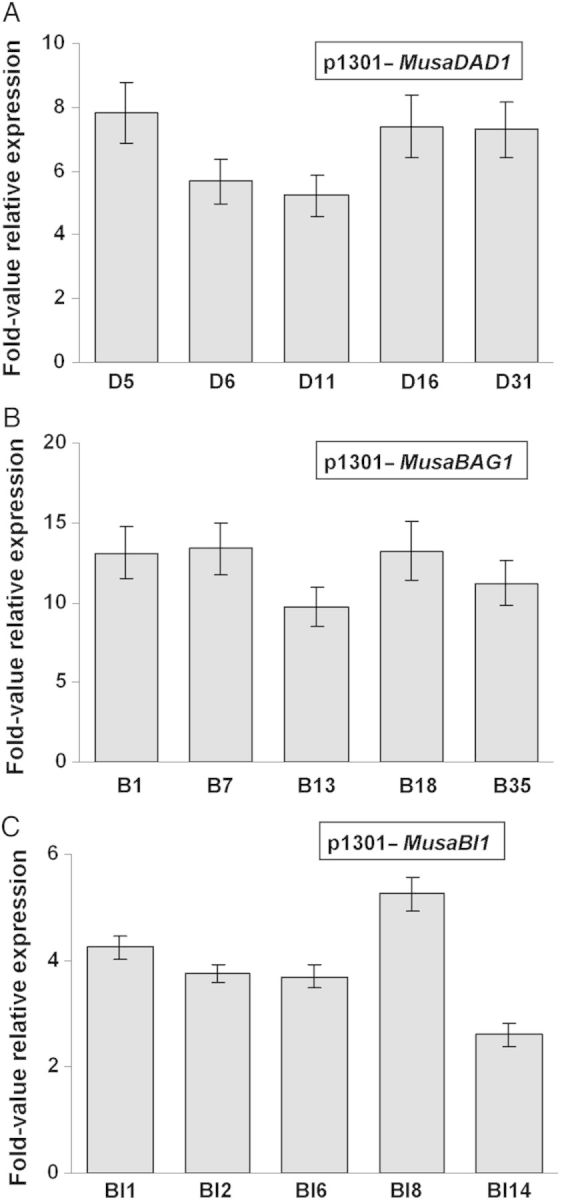
Fold-value overexpression analysis of transgenic banana plants. The exact quantum of overexpression of *MusaDAD1*, *MusaBAG1* and *MusaBI1* in transgenic banana lines was determined using real-time quantitative RT–PCR. The *x*-axis represents the expression level of native *MusaDAD1*, *MusaBAG1* and *MusaBI1* in untransformed control plants. Values are the mean ± SE.

Real-time quantitative RT–PCR assays performed using cDNAs derived from the same selected transgenic lines showed efficient overexpression of the three genes (*MusaDAD1*, *MusaBAG1* and *MusaBI1*) in the respective transgenic lines as compared with the untransformed control plants (Fig. 6). Transgenic lines derived from each construct differed in the level of overexpression of the three genes probably due to the difference in the T-DNA copy numbers and their site of integration in the banana genome. Further, the maximum overexpression was noticed in *MusaBAG1* overexpressing plants probably explaining the best resistance against Foc observed in these plants.

## Discussion

Owing to the recent spread of highly virulent Foc Race 4 beyond South-East Asia and Australia, the threat of a major disease epidemic in major banana-growing areas of the world, mainly Africa and Asia has become very real. Genetic engineering of elite banana cultivars for resistance to Fusarium wilt is the only pragmatic goal as there are no established Foc Race 4 cultivars readily available right now. In the present study, three genes putatively involved in cell-death-related processes in banana (*MusaDAD1*, *MusaBAG1* and *MusaBI1*) were identified and overexpressed in transgenic banana plants. The plants overexpressing *MusaBAG1* demonstrated the best resistance towards Foc infection in greenhouse bioassays.

Several different strategies have been employed in the past for development of Foc resistance in banana with varied success. Overexpression of anti-microbial proteins like magainin (Chakrabarti *et al.* 2003) and defensins (Mohandas *et al.* 2011; Ghag *et al.* 2012) in transgenic banana plants has led to some level of tolerance to Fusarium wilt. Other groups have tried animal cells derived apoptosis-inhibition-related genes namely *CED9*, *Bcl-xL* and *Bcl-2 3*′ *UTR* to prevent the necrotropic death of banana plants after the Foc establishes itself in the host plant (Paul *et al.* 2011). Apart from *Fusarium*, apoptosis-inhibition-related genes have also been employed in the control of Verticillium wilt in cotton and against different pathogens in tomato (Lincoln *et al.* 2002; Tian *et al.* 2010).

Since there are multiple confirmed reports describing the manipulation of host PCD machinery by an invading fungal pathogen in plants (Govrin and Levine 2000; Sharon and Finkelshtein 2009; Dickman and de Figueiredo 2011), a detailed study of the genes involved in UPR response and the PCD pathway in plants is warranted. Since Foc is considered to be a hemibiotrophic pathogen which involves a long saprophytic phase on the banana plant, investigations into the genes related with PCD are necessary to fully understand the progression of this disease in banana. Accordingly, three such genes (*MusaDAD1*, *MusaBAG1* and *MusaBI1*) were identified in banana by homology searching (using genes derived from rice and *Arabidopsis* as query) in the EST databases of banana. Expression profiling of these genes in banana cells treated with known Foc derived toxins (FA and BEA) or the active culture/autoclaved filtrate (aFoc/fFoc) indicated differential regulation of the three genes in response to these four treatments. The fact that BEA, aFoc and fFoc treatments led to similar expression profiles for all the three genes studied indicated that they probably activated common signalling pathways upon inoculation. Further, since these effects were reported in just 6 h post-inoculation, this suggested that BEA probably acts in the initial stages of the infection process. Also, the fact that *MusaBAG1* was induced to significant levels in BEA-treated cells (similar to those which were treated with aFoc and fFoc) correlated well with our later observations wherein transgenic banana plants overexpressing *MusaBAG1* (at levels which were several folds higher than its induction in response to BEA or the aFoc/fFoc treatments) were found to be highly resistant to Foc Race 1 infection. Conversely, FA induced the three genes studied in a diagonally inverse pattern as compared with the other three treatments. Fusaric acid, which down-regulated *MusaBAG1* in the treated cells, therefore, appears to work towards the induction of the PCD and thereby the necrotropic phase of Foc life cycle on the host banana plant. Although FA induced the expression of *MusaBI1* to significant levels, the fact that *MusaBI1* overexpressing plants did not show impressive Foc resistance indicated that unlike *MusaBAG1*, the role of *MusaBI1* in the establishment of Foc infection on host banana plants is not very significant. As regards the *MusaDAD1* gene regulation, although we could not find any significant change in the expression level of this gene in three of four treatments preformed, past reports have shown significant down-regulation of this gene in Foc susceptible as well as resistant plants upon inoculation with Foc spores (Li *et al.* 2012). Several reports in other plants have demonstrated that similar *DAD* genes prevent the onset of PCD in plants (Danon *et al.* 2004). Thus, although the role played by *DAD* homologues in PCD and Foc disease progression is still not clear, we did notice some level of resistance to Foc (although corm regions appear discoloured, the aerial portions of the banana plant were relatively symptom less) in *MusaDAD1* overexpressing plants.

Among the three genes studied as part of this study, we could get the maximum relative overexpression in *MusaBAG1* overexpressing plants. This was probably the result of lower steady state levels of the *MusaBAG1* gene in uninfected untransformed control plants. Concurrently, the best resistance against Foc was also noticed in *MusaBAG1* overexpressing plants. So, although all the three genes studied here have been associated with improved resistance against specific pathogens in model plants like *Arabidopsis*, we propose a far greater role for the *MusaBAG1* gene in the control of PCD in banana plant as compared with the other two genes studied. Further, in the face of stiff resistance towards development of novel transgenic plants with genes derived from animal systems (and thereby allegedly crossing nature barriers), use of native genes like *MusaBAG1* for incorporating efficient resistance towards pathogens like Foc is expected to be far more acceptable to the scientific community as well as to the general populace. These plants can potentially resist Foc Race 4 infection also. But since Foc Race 4 has still not been reported from India (and any such effort to import Foc Race 4 spores by India risks its accidental spread there), no bioassay against Foc Race 4 could be performed with the plants developed during this study.

In conclusion, use of native genes like *MusaBAG1* for development of Fusarium wilt resistance in cultivars like *Rasthali* could be the best way to improve the production of local cultivars where huge losses have been reported due to Fusarium wilt in the past.

## Sources of Funding

This work was funded by the Department of Atomic Energy, Government of India.

## Contributions by the Authors

S.B.G., U.K.S.S. and T.R.G. conceived and designed the experiments and wrote the paper; S.B.G. performed the experiments; U.K.S.S. and S.B.G. analysed the data; and T.R.G. and U.K.S.S. contributed reagents/materials/analysis tools.

## Conflicts of Interest Statement

None declared.

## Accession Numbers

The nucleotide sequences of *MusaDAD1*, *MusaBAG1* and *MusaBI1* from banana have been submitted to GenBank with accession numbers KJ636052, KJ636053 and KJ636054, respectively.

## Supporting Information

The following Supporting Information is available in the online version of this article –

**Table S1.** Primers used in the present study.

**Fig. S1.** Sequence and phylogenetic analysis of *MusaDAD1.*

**Fig. S2.** Sequence and phylogenetic analysis of *MusaBAG1*.

**Fig. S3.** Sequence and phylogenetic analysis of *MusaBI1*.

Additional Information

## References

[PLU037C1] Buddenhagen IW (2009). Understanding strain diversity in *Fusarium oxysporum* f. sp. *cubense* and history of the introduction of ‘Tropical race 4’ to better manage banana production.

[PLU037C2] Butler D (2013). Fungus threatens top banana: fears rise for Latin American industry as devastating disease hits leading variety in Africa and Middle East. Nature.

[PLU037C3] Chakrabarti A, Ganapathi TR, Mukherjee PK, Bapat VA (2003). MSI-99, a magainin analogue, imparts enhanced disease resistance in transgenic tobacco and banana. Planta.

[PLU037C4] Danon A, Rotari VI, Gordon A, Mailhac N, Gallois P (2004). Ultraviolet-C overexposure induces programmed cell death in *Arabidopsis*, which is mediated by caspase-like activities and which can be suppressed by caspase inhibitors, p35 and Defender against apoptopic death. The Journal of Biological Chemistry.

[PLU037C5] Dickman MB, de Figueiredo P (2011). Comparative pathobiology of fungal pathogens of plants and animals. PLoS Pathogens.

[PLU037C6] Dickman MB, de Figueiredo P (2013). Death be not proud—cell death control in plant fungal interactions. PLoS Pathogens.

[PLU037C7] Dickman MB, Park YK, Oltersdorf T, Li W, Clemente T, French R (2001). Abrogation of disease development in plants expressing animal antiapoptotic genes. Proceedings of the National Academy of Sciences of the USA.

[PLU037C8] Dong YH, Zhan XC, Kvarnheden A, Atkinson RG, Morris BA, Gardner RC (1998). Expression of a cDNA from apple encoding a homologue of DAD1, an inhibitor of programmed cell death. Plant Science.

[PLU037C9] Doukhanina EV, Chen S, Zalm E, Godzik A, Reed J, Dickman MB (2006). Identification and functional characterization of the BAG protein family in *Arabidopsis thaliana*. The Journal of Biological Chemistry.

[PLU037C11] Ganapathi TR, Higgs NS, Balint-Kurti PJ, Arntzen CJ, May GD, Van Eck JM (2001). *Agrobacterium*-mediated transformation of the embryogenic cell suspensions of the banana cultivar *Rasthali* (AAB). Plant Cell Reports.

[PLU037C12] Ghag SB, Shekhawat UKS, Ganapathi TR (2012). Petunia floral defensins with unique prodomains as novel candidates for development of Fusarium wilt resistance in transgenic banana plants. PLoS ONE.

[PLU037C13] Ghag SB, Shekhawat UKS, Ganapathi TR (2014). Host induced post-transcriptional hairpin RNA-mediated gene silencing of vital fungal genes confers efficient resistance against Fusarium wilt in banana. Plant Biotechnology Journal.

[PLU037C14] Govrin EM, Levine A (2000). The hypersensitive response facilitates plant infection by the necrotrophic pathogen *Botrytis cinerea*. Current Biology.

[PLU037C16] Heslop-Harrison JS, Schwarzacher T (2007). Domestication, genomics and the future for banana. Annals of Botany.

[PLU037C17] Hückelhoven R, Dechert C, Kogel KH (2003). Overexpression of barley BAX inhibitor 1 induces breakdown of Mlo-mediated penetration resistance to *Blumeria graminis*. Proceedings of the National Academy of Sciences of the USA.

[PLU037C18] Hwang SC, Ko WH (2004). Cavendish banana cultivars resistant to Fusarium wilt acquired through somaclonal variation in Taiwan. Plant Disease.

[PLU037C19] Jones DR (2009). Disease and pest constraints to banana production. ISHS Acta Horticulturae.

[PLU037C20] Lam E, Kato N, Lawton M (2001). Programmed cell death, mitochondria and the plant hypersensitive response. Nature.

[PLU037C22] Li C, Chen S, Zuo C, Sun Q, Ye Q, Yi G, Huang B (2011). The use of GFP-transformed isolates to study infection of banana with *Fusarium oxysporum* f. sp. *cubense* race 4. European Journal of Plant Pathology.

[PLU037C23] Li CY, Deng GM, Yang J, Viljoen A, Jin Y, Kuang RB, Zuo CW, Lv ZC, Yang QS, Sheng O, Wei YR, Hu CH, Dong T, Yi GJ (2012). Transcriptome profiling of resistant and susceptible Cavendish banana roots following inoculation with *Fusarium oxysporum* f. sp. *cubense* tropical race 4. BMC Genomics.

[PLU037C24] Lincoln JE, Richael C, Overduin B, Smith K, Bostock R, Gilchrist DG (2002). Expression of the antiapoptotic baculovirus p35 gene in tomato blocks programmed cell death and provides broad-spectrum resistance to disease. Proceedings of the National Academy of Sciences of the USA.

[PLU037C25] Liua JX, Howell SH (2010). Endoplasmic reticulum protein quality control and its relationship to environmental stress responses in plants. The Plant Cell.

[PLU037C26] Matsumura H, Nirasawa S, Kiba A, Urasaki N, Saitoh H, Ito M, Kawai-Yamada M, Uchimiya H, Terauchi R (2003). Overexpression of Bax inhibitor suppresses the fungal elicitor-induced cell death in rice (*Oryza sativa* L.) cells. The Plant Journal.

[PLU037C27] Mitsuhara I, Malik KA, Miura M, Ohashi Y (1999). Animal cell-death suppressors Bcl-xL and Ced-9 inhibit cell death in tobacco plants. Current Biology.

[PLU037C28] Mohandas S, Manjula R, Saxena AK, Ajay KM, Shakunthala B, Sowmya HD, Meenakshi S (2011). Transformation of the banana cultivar ‘Nanjangud Rasbale’ (syn. ‘Rasthali’, aab, silk subgroup) with the amp gene and screening for *Fusarium* resistance with a bioassay. ISHS Acta Horticulturae.

[PLU037C29] Paul JY, Becker DK, Dickman MB, Harding RM, Khanna HK, Dale JL (2011). Apoptosis-related genes confer resistance to Fusarium wilt in transgenic ‘Lady Finger’ bananas. Plant Biotechnology Journal.

[PLU037C30] Pfaffl MW, Horgan GW, Dempﬂe L (2002). Relative expression software tool (REST) for group-wise comparison and statistical analysis of relative expression results in real-time PCR. Nucleic Acids Research.

[PLU037C31] Ploetz RC (2006). Fusarium wilt of banana is caused by several pathogens referred to as *Fusarium oxysporum* f. sp. *cubense*. Phytopathology.

[PLU037C32] Sharon A, Finkelshtein A, Esser K, Deising H (2009). Programmed cell death in fungus–plant interactions. The mycota.

[PLU037C33] Shekhawat UKS, Srinivas L, Ganapathi TR (2011). *MusaDHN-1*, a novel multiple stress-inducible SK(3)-type dehydrin gene, contributes affirmatively to drought- and salt-stress tolerance in banana. Planta.

[PLU037C34] Sreedharan S, Shekhawat UKS, Ganapathi TR (2013). Transgenic banana plants overexpressing a native plasma membrane aquaporin *MusaPIP1;2* display high tolerance levels to different abiotic stresses. Plant Biotechnology Journal.

[PLU037C35] Tang CY, Chang WC, Drew R (2005). Seasonal variation: a tool for the improvement of Cavendish banana cultivars. Proceedings of the Kind IS on Biotech. of tropical and subtropical species.

[PLU037C36] Thaler JS, Owen B, Higgins VJ (2004). The role of the jasmonate response in plant susceptibility to diverse pathogens with a range of lifestyles. Plant Physiology.

[PLU037C37] Tian J, Zhang X, Liang B, Li S, Wu Z, Wang Q, Leng C, Dong J, Wang T (2010). Expression of Baculovirus anti-apoptotic genes p35 and op-iap in Cotton (*Gossypium hirsutum* L.) enhances tolerance to Verticillium wilt. PLoS ONE.

[PLU037C38] Wang XJ, Tang CL, Huang XL, Li FF, Chen XM, Zhang G, Sun YF, Han DJ, Kang ZS (2012). Wheat BAX inhibitor-1 contributes to wheat resistance to *Puccinia striiformis*. Journal of Experimental Botany.

[PLU037C39] Watanabe N, Lam E (2008). BAX inhibitor-1 modulates endoplasmic reticulum stress-mediated programmed cell death in *Arabidopsis*. The Journal of Biological Chemistry.

[PLU037C40] Williams B, Kabbage M, Britt R, Dickman MB (2010). AtBAG7, an *Arabidopsis* Bcl-2-associated athanogene, resides in the endoplasmic reticulum and is involved in the unfolded protein response. Proceedings of the National Academy of Sciences of the USA.

[PLU037C41] Yoshikawa M, Matama M, Masago H (1981). Release of a soluble phytoalexin elicitor from mycelial walls of *Phytophthora megasperma* var. *sojae* by soybean tissues. Plant Physiology.

