# Supplementary material for: Native cell-death genes as candidates for developing wilt resistance in transgenic banana plants
Source: AoB Plants. 2014 Jul 4;6:plu037. doi: 10.1093/aobpla/plu037 (PMC4122335; doi:10.1093/aobpla/plu037)
Supplement: Additional Information [file supp_6_plu037_index.html]

Native cell-death genes as candidates for developing wilt resistance in transgenic banana plants — Additional Information 

# Native cell-death genes as candidates for developing wilt resistance in transgenic banana plants

## Additional Information

Additional Information

**Files in this Data Supplement:**

- Additional Information - doc file
- Additional Information Figure 1 - jpg file
- Additional Information Figure 2 - jpg file
- Additional Information Figure 3 - jpg file
