# Supplementary material for: Native cell-death genes as candidates for developing wilt resistance in transgenic banana plants
Source: AoB Plants. 2014 Jul 4;6:plu037. doi: 10.1093/aobpla/plu037 (PMC4122335; doi:10.1093/aobpla/plu037)
Supplement: Additional Information [file supp_plu037_plu037supp.doc]

**Table S1: Primers used in the present study**

| **PCR Reaction** |  | **Primer Sequence (5’-3’)** |
| --- | --- | --- |
| Amplification of complete coding sequence of *MusaDAD1* | Fw | AACTCTGCAGATGGCAAAGTCAAATGCAAGT |
|  | Rv | AGGTGGTACCAACTAGCTAGCCGAGGAAGTTCA |
| Amplification of complete coding sequence of *MusaBAG1* | Fw | AACTCTGCAGATGATCAGGTTGAGATCAAAGAA |
|  | Rv | AGGTGGTACCAAGGCCTACACCTCAATAATGG |
| Amplification of complete coding sequence of *MusaBI1* | Fw | ATGTCCTGCAGGATGGATTCCTTCTTCCAATCTC |
|  | Rv | CATCGGTACCCCAGGATTTTACGATCTCTTCTTT |
| Real time quantitative amplification of *MusaDAD1* | Fw | TTCACTGCGCTCAGCTTATG |
|  | Rv | AGCCCGTTCAGGAGGTAAAT |
| Real time quantitative amplification of *MusaBAG1* | Fw | GAGGGAGCTGATGAGGTGAT |
|  | Rv | AAACCAGCACCTTGTCCTTG |
| Real time quantitative amplification of *MusaBI1* | Fw | TGACGCTATGTTGTGCCCTA |
|  | Rv | TCGGGTCAAAGTCAATAGCC |
| Real time quantitative amplification of *Musa* *EF1α* | Fw | CCGATTGTGCTGTCCTCATT |
|  | Rv | TTGGCACGAAAGGAATCTTCT |
| Amplification of nos 3’ UTR | Fw | CTACCGAGCTCGAATTTCCCCGATCGTT |
|  | Rv | CGGCCAGTGAATTCCCGATCTAGTAACA |
| Amplification of *Zea mays* polybuiquitin promoter | Fw | aattAAGCTTccggtcgtgcccctctcta |
|  | Rv | agctctgcagaagtaacaccaaacaacagg |
| Amplification of *hygromycin phosphotransferase* gene | Fw | GTCCTGCGGGTAAATAGCTG |
|  | Rv | ATTTGTGTACGCCCGACAGT |
