# Supplementary figures and images for: Native cell-death genes as candidates for developing wilt resistance in transgenic banana plants
Source: AoB Plants. 2014 Jul 4;6:plu037. doi: 10.1093/aobpla/plu037 (PMC4122335; doi:10.1093/aobpla/plu037)

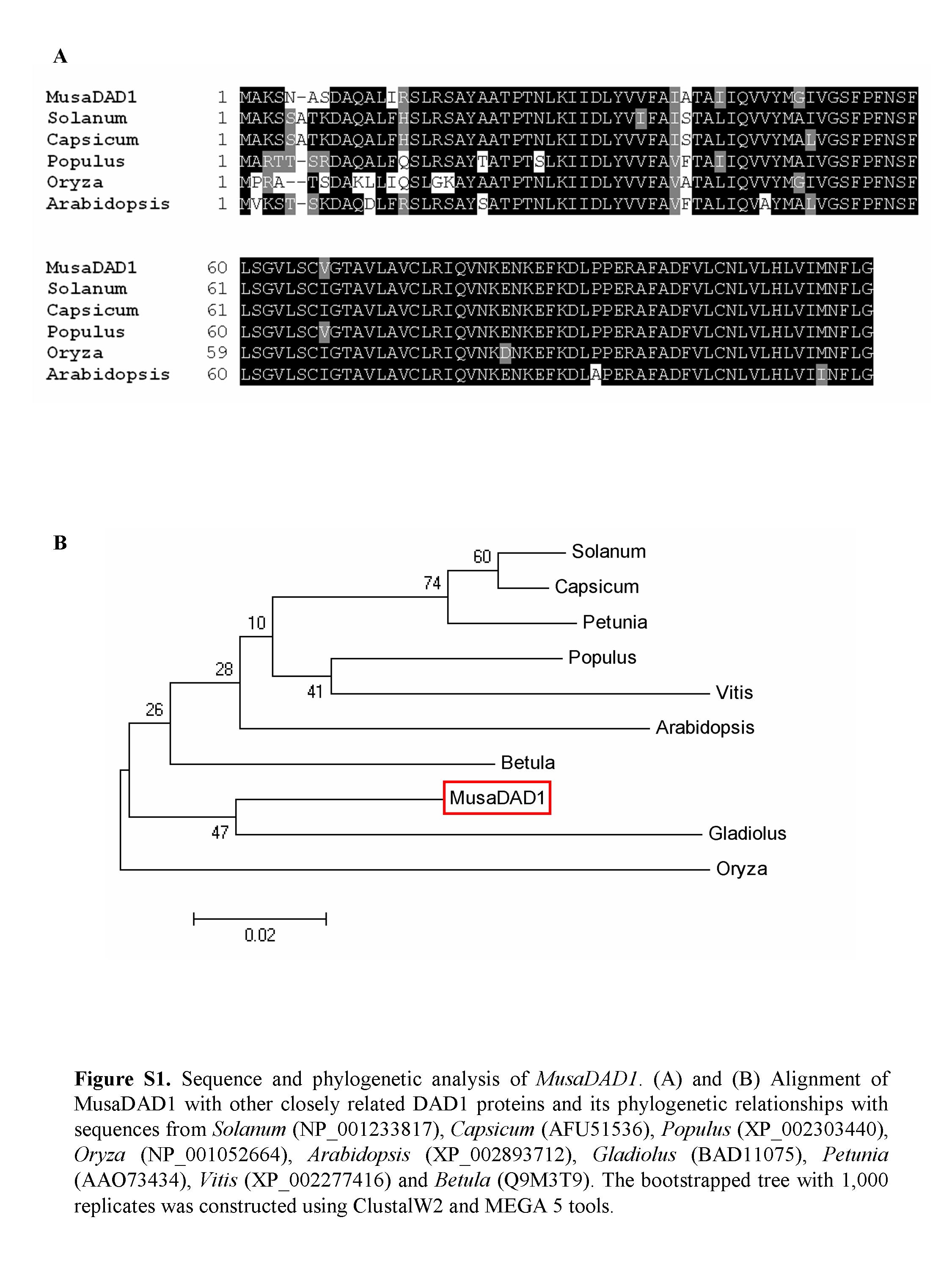

Supplement: Additional Information [file supp_plu037_plu037supp_fig1.jpg]

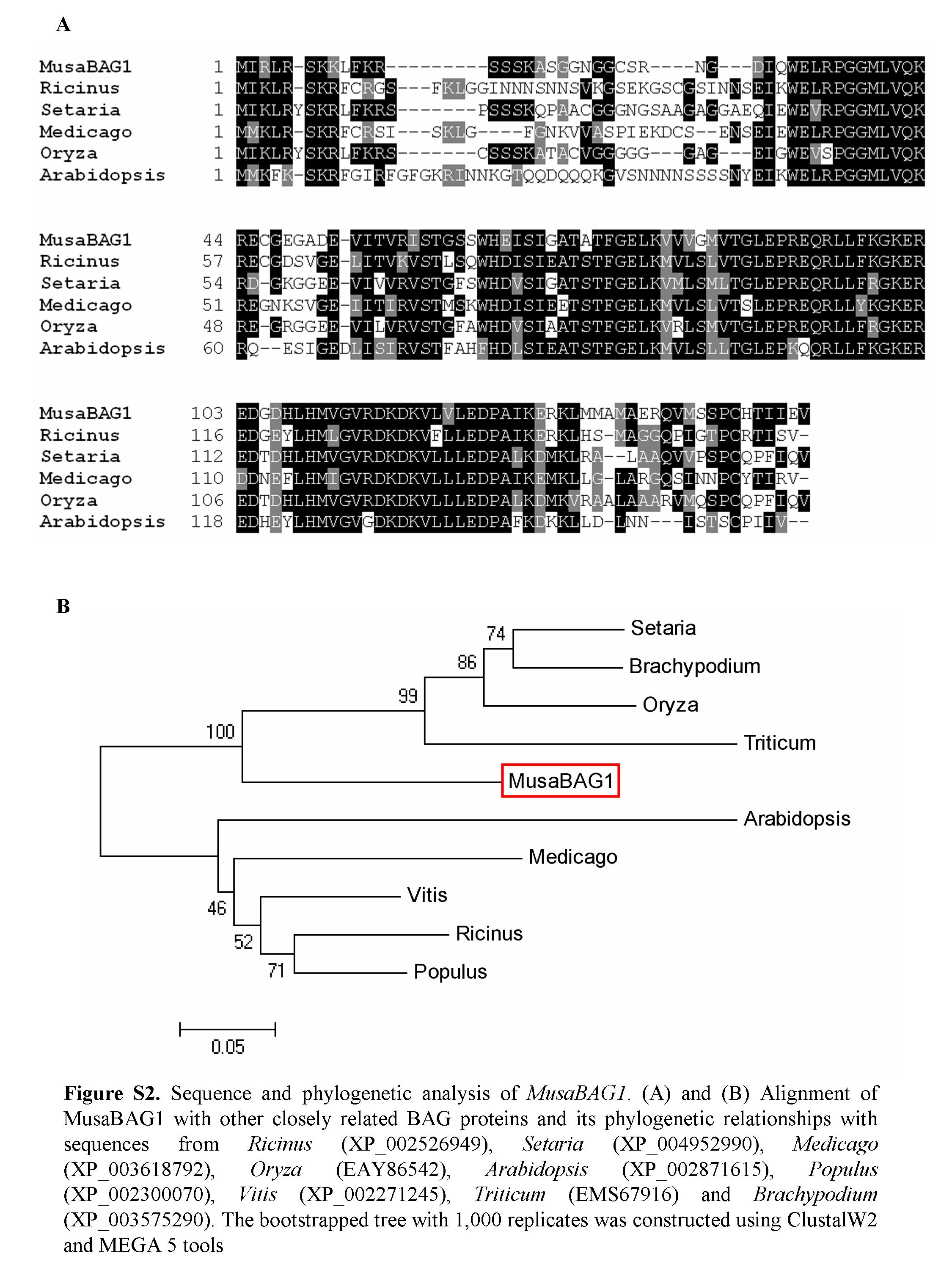

Supplement: Additional Information [file supp_plu037_plu037supp_fig2.jpg]

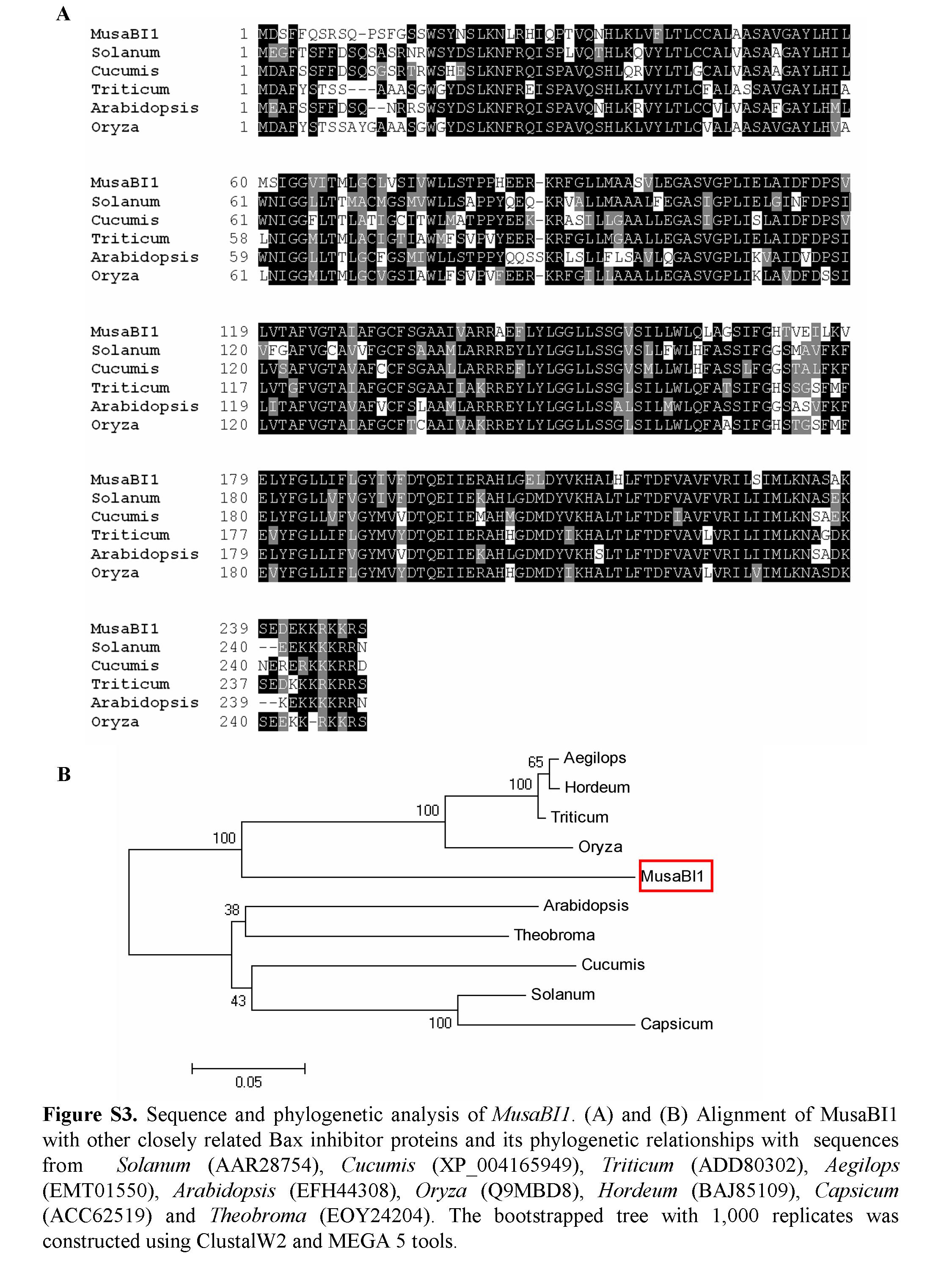

Supplement: Additional Information [file supp_plu037_plu037supp_fig3.jpg]
